# Cleidocranial dysplasia

**DOI:** 10.4103/0970-2113.68322

**Published:** 2010

**Authors:** Ramakant Dixit, Kalpana Dixit, A. R. Paramez

**Affiliations:** *Department of Pulmonary Medicine, JLN Medical College, Ajmer and Samarpan Child Clinic, Taragarh Link Road, Ajmer, India*

**Keywords:** Clavicle hypoplasia, cleidocranial dysplasia, genetic disorder

## Abstract

Cleidocranial dysplasia is a rare autosomal dominant condition with generalized dysplasia of bone, characterized by delayed closer of cranial sutures, hypoplastic or aplastic clavicles, short stature, dental abnormalities and a variety of other skeletal abnormalities. We present a seven-year-old female child presenting with classical features of cleidocranial dysplasia.

## INTRODUCTION

Cleidocranial dysplasia (CCD) is a rare disorder of autosomal dominant inheritance characterized by delayed closure of the cranial sutures, hypoplastic or aplastic clavicles and multiple dental abnormalities.[1] This condition is usually caused by a mutation of the RUNX_2_ (Core Binding Factor-α_1_) gene, located at chromosome 6p21. This gene encodes a protein necessary for the correct functioning of osteoblast cells.[2] However, 40% of the cases of CCD appear spontaneously with no apparent genetic cause. CCD affects most prominently those bones derived from endochondral and intramembranous ossification, such as the cranium and the clavicles. The diagnosis is based on clinical and radiological findings.[3] The present communication describes a case of CCD.

## CASE REPORT

A seven-year-old female child presented with proportionate short stature (height 82cm, upper segment to lower segment ratio 4:5), delayed mile stones and history of fracture on minor trauma. On examination, she had dismorphic features i.e. macrocephaly (head circumference 50 cm), frontal and parietal bossing, open sutures with wide open anterior fontanels, slightly bluish sclera, depressed nasal bridge, low set ears, high arched palate, delayed tooth eruption, long neck, narrow sloping shoulders that could be approximated anteriorly. She also had slight lordosis, bowing of limbs, flat foot and mild hypotonia. Her intelligence was absolutely normal and other systemic examination was unremarkable.

**Figure 1:**
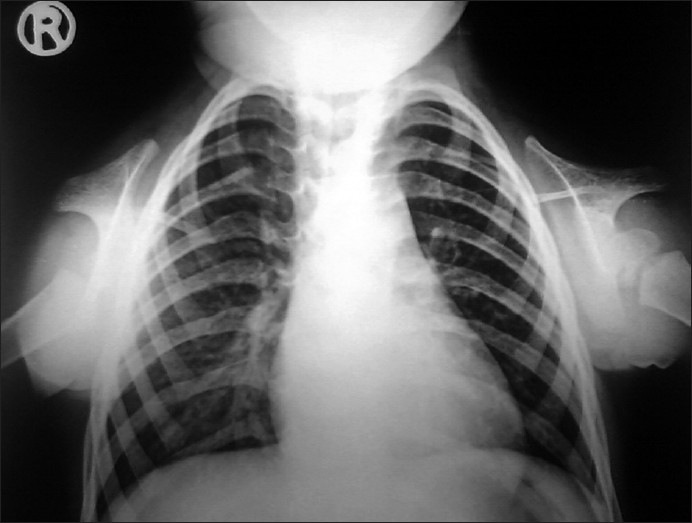
X-ray chest showing bilateral, severely hypoplastic clavicles seen only in lateral aspects

**Figure 2:**
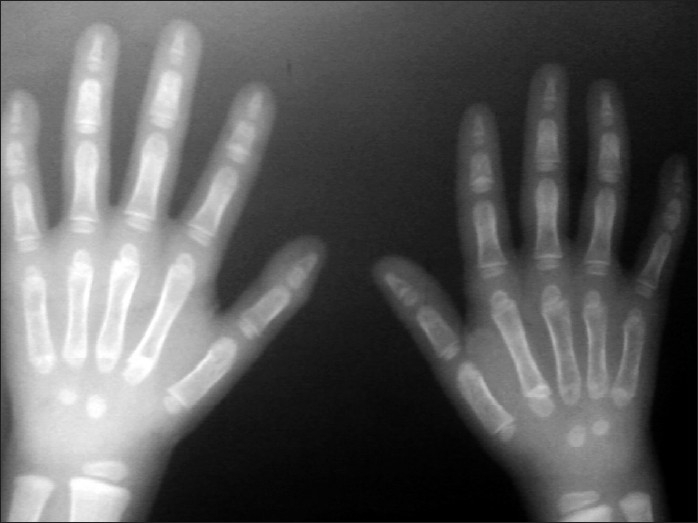
X-ray hand showing only two carpal bones

Her investigations revealed normal hemoglobin, blood cell counts and blood biochemistry including liver function tests, renal function tests, electrolytes; 24-hour urine examination for calcium, phosphorus and creatinine were also normal. Stool examination showed cyst of Giardia. Her thyroid profile, growth hormone assay and tissue trans-glutaminase antibody (IgA) level were also normal. Skeletal survey revealed bilateral severely hypoplastic clavicles seen only in lateral parts [Figure 1], barrel shaped chest, spina bifida at upper dorsal vertebrae, only two carpal bones seen in both hands [Figure 2] and no abnormalities in x-rays of other bony parts. The above clinical and radiological features were suggestive of CCD. Patient is the only child of non-consanguineous marriage and family history of both maternal and paternal side did not reveal any such malformation.

## DISCUSSION

CCD is a relatively uncommon disorder with a prevalence of 0.5 per 100,000 live births.[4] Clinically, the diagnosis is often made at birth but may not occur until later, when persistence of the widely open anterior fontanels and sutures or short stature incites parental concern. Individuals with this disorder present with some or all of very characteristic features. These include large brachycephalic head, small and angular face, prominent frontal and parietal bones and drooping shoulder with excessive mobility. Depending on the amount of clavicular involvement, the patient may be able to approximate the shoulders anteriorly.[3] Height is reduced in both sexes and chest may be narrowed or funnel-shaped leading to potential respiratory distress in childhood. Abnormal dentition, including delayed eruption of secondary dentition, failure to shed the primary teeth, variable numbers of supernumerary teeth along with dental crowding and malocclusion are common findings. Other facial features include broad base of the nose, depressed nasal bridge, narrow high-arched palate, absent paranasal sinuses and hypertelorism. Hand abnormalities may be brachydactyly, tapering fingers and short, broad thumbs. The patient’s cognitive abilities are normal in typical cases of CCD.[4–6] Most of these features were seen in the present case.

The radiological features of CCD are also very characteristic. The cranial abnormalities include wide-open sutures, patent fontanels, presence of wormian bones, and delayed ossification of skull, poor/absent pneumatization of paranasal, frontal and mastoid sinuses, and impacted, crowded and supernumerary teeth. Chest X-ray shows cone shaped thorax with narrow upper thoracic diameter, complete absence or hypoplastic clavicles and hypoplastic scapulae etc. X-ray pelvis may show delayed ossification with wide pubic symphysis, hypoplastic iliac wings, widened sacroiliac joints and large femoral neck etc.

X-ray hand shows pseudoepiphyses of the metacarpal and metatarsal bones resulting in characteristic lengthening of the second metacarpal, hypoplastic and pointed digital phalanges etc. Spinal changes include spina bifida occulta in the cervical and upper thoracic region. The vertebrae may show delayed mineralization.[4,7–9] Many of these features were evident in the present case also.

The differential diagnosis of CCD includes Crane-Heise syndrome, mandibuloacral dysplasia, pycnodysostosis, yunis varon syndrome, CDAGS syndrome and hypophosphatasia etc.[1] These conditions may share some characteristics with CCD, however all these are autosomal recessive disorders and have other specific features. Some of these conditions may result from mutation in genes that affect the action of RUNX_2_ on its downstream targets.[10]

Common complications of CCD include pes planus, genu velgum, shoulder and hip dislocation, recurrent sinus infections, upper airway complications, recurrent ear infection, hearing loss, dental caries, osteomyelitis of the mandible or maxilla, respiratory distress in early infancy etc.[1,4,5] However, even with these potential complications, the life span in such patients is normal.
